# Gut microbiota in patients with Alzheimer’s disease spectrum: a systematic review and meta-analysis

**DOI:** 10.18632/aging.203826

**Published:** 2022-01-14

**Authors:** Chun-Che Hung, Chiung-Chih Chang, Chi-Wei Huang, Rui Nouchi, Chia-Hsiung Cheng

**Affiliations:** 1 Department of Occupational Therapy and Graduate Institute of Behavioral Sciences, Chang Gung University, Taoyuan, Taiwan; 2 Laboratory of Brain Imaging and Neural Dynamics (BIND Lab), Chang Gung University, Taoyuan, Taiwan; 3 Department of Neurology and Institute for Translational Research in Biomedicine, Kaohsiung Chang Gung Memorial Hospital, Chang Gung University College of Medicine, Kaohsiung, Taiwan; 4 Department of Cognitive Health Science, Institute of Development, Aging and Cancer (IDAC), Tohoku University, Sendai, Japan; 5 Smart Aging Research Center (S.A.R.C), Tohoku University, Sendai, Japan; 6 Healthy Aging Research Center, Chang Gung University, Taoyuan, Taiwan; 7 Department of Psychiatry, Chang Gung Memorial Hospital, Linkou, Taiwan

**Keywords:** Alzheimer’s disease, mild cognitive impairment, gut microbiota, dysbiosis, systematic review, meta-analysis

## Abstract

Context: Gut dysbiosis has been proposed as one of pathologies in patients with Alzheimer’s disease (AD) spectrum. Despite such enthusiasm, the relevant results remain substantially controversial.

Objective: A systematic review and meta-analysis were performed to investigate the differences of gut microbiota (GM) between patients with AD spectrum (including mild cognitive impairment [MCI] and AD) and healthy controls (HC).

Data sources: PubMed, MEDLINE, Scopus, and Cochrane Library from January 2000 to August 2021.

Eligibility criteria for study selection: Observational trials and pre-intervention data of intervention trials that investigated the abundance of GM in patients with AD spectrum and HC.

Data extraction and synthesis: Two reviewers independently identified articles, extracted data, and evaluated the risk of bias. The effect sizes were performed by a random-effect, inverse-variance weighted model. The effects of different countries and of clinical stages on GM abundance were also examined.

Results: 11 studies consisting of 378 HC and 427 patients with AD spectrum were included in the meta-analysis. Patients with AD, but not MCI, showed significantly reduced GM diversity as compared to HC. We also found more abundance of *Proteobacteria*, *Bifidobacterium* and *Phascolarctobacterium*, but less abundance of *Firmicutes*, *Clostridiaceae*, *Lachnospiraceae* and *Rikenellaceae* in patients with AD spectrum as compared with HC. The profiles of abundance of *Alistipes* and *Bacteroides* in HC and AD spectrum were differentially affected by countries. Finally, when considering clinical stage as a moderator, the comparisons of abundance in *Clostridiaceae* and *Phascolarctobacterium* showed large effect sizes, with gradient changes from MCI to AD stage.

Limitations: The inclusion of studies originating only from China and the U.S. was a possible limitation.

Conclusions: Patients with AD spectrum demonstrated altered GM abundance, which was differentially mediated by countries and clinical stages.

## INTRODUCTION

Previous studies have suggested that amyloid-beta (Aβ) peptide deposition in the brain is an early neural change in patients with Alzheimer’s disease (AD) [1, 2]. However, the etiopathogenesis of AD are not well explained. Recent evidence has focused on a potential role of gut microbiota (GM) in the development or exacerbation of AD [3–5].

There are thousands of microbes residing in the human gut, which involves crucial functions for individual physiology and development [6]. Moreover, accumulating evidence has revealed that the gut and central nervous system (CNS) interact with one another through the following neuro-chemical pathways. First, GM may produce and release neurotransmitters and neurotoxins such as short-chain fatty acids (SCFAs), 5HT, acetylcholine, tryptophan, and D-lactate and ammonia [7–9]. All these molecules are transmitted by the systemic circulation and then cross the blood-brain barrier (BBB) to modulate neural activities. Second, connections of enteric nervous system (ENS) and CNS is through the vagus nerve and the autonomic nervous system [10]. Upon activation of ENS, it receives signals from GM, and then affects the gut cells and regulates anti-inflammatory effects of the peripheral immune system [11, 12]. Finally, GM is involved in the modulation of immune system through the synthesis and release of pro-inflammatory cytokines such as interleukin-1, interleukin-6 and tumor necrosis factor-alpha [13, 14]. Interestingly, previous studies have found that GM affects the host’s maturation of the neuroendocrine, nervous, and immune system; hence, the gut-brain axis plays an important role in the bidirectional communications between the ENS and CNS [15–17]. Notably, compelling evidence has proposed that any disturbance in these routes would potentially be associated with the AD occurrence [18, 19].

More recently, the changes in diversity and equilibrium of GM have attracted much attention in many neurological and psychiatric disorders. When the intestinal ecosystem is abnormally altered, the composition of GM becomes imbalanced (i.e., dysbiosis). This dysbiotic pattern prompts the host to establish a disease-related microbial community, leading to leaky intestine and BBB, as well as bacterial translocation [20]. Animal studies have demonstrated that gut dysbiosis is involved in the pathogenesis of AD [21, 22]. Studies from clinical settings have also explored the composition of GM in the patients with AD spectrum, including mild cognitive impairment (MCI) and AD [23–28]. Several GM strains were reported to be associated with the cognitive functions and neuropsychiatric symptoms in patients with AD [29]. Furthermore, it has been suggested that probiotics supplementation may be an effective dietary intervention for individuals with AD [30, 31] and other conditions, such as polycystic ovarian syndrome [32] and major depressive disorder [33].

It is interesting to note that the composition of GM is distinct from country to country. For instance, a previous study from U.S. showed an alteration of GM in patients with AD, comprising increased *Bacteroidetes* and reduced *Actinobacteria* in the phylum level [26]. In contrast, Zhuang and colleagues demonstrated opposite results in Chinese patients with AD (i.e., reduced *Bacteroidetes* and increased *Actinobacteria*) [27]. In addition, the magnitudes of gut dysbiosis have been reported to be different between patients with MCI and AD. Most of the existing literature revealed that patients with AD, but not MCI, demonstrated significantly reduced GM diversity compared to healthy older adults [25, 26, 29]. However, there was a study reporting similar GM diversity and abundance in patients with MCI and AD [24]. Thus, it remains unclear whether different clinical stages lead to different magnitudes of gut dysbiosis.

To the best of our knowledge, no statistical review of GM structure in patients with AD spectrum has been performed. Therefore, the purpose of this study was two-fold. First, we aimed to determine the differences of GM diversity and abundance between the patients with AD spectrum and healthy controls (HC). Second, we further examined the potential effects of different countries and clinical stages on GM abundance.

## METHODS

### Literature search

This meta-analysis followed the Preferred Reporting Items for Systematic reviews and Meta-Analyses (PRISMA) guidelines [34]. We conducted a comprehensive literature search in PubMed, MEDLINE, Scopus, and Cochrane Library electronic databases from January 2000 to August 2021, with combinations of the following terms: (“Alzheimer’s disease” OR “dementia” OR “mild cognitive impairment” OR “cognitive dysfunction”) AND (“microbiota” OR “gut microbiota” OR “microbiome”). Moreover, the reference lists of the selected articles or reviews were also included as additional studies.

### Eligibility criteria

Two authors (CCH and CHC) independently screened and identified the full texts that met the following inclusion criteria: (1) they were peer-reviewed articles written in English; (2) GM diversity and abundance was compared between patients with AD spectrum and HC; (3) GM was derived from stool samples; (4) only pre-intervention data were collected from the intervention studies; (5) the GM strains were investigated by at least three studies; (6) adequate statistical data (e.g., mean, standard deviation, *p* values, median, maximum, minimum, etc.) to estimate effect sizes. Studies of case reports, systematic reviews and animal research were excluded.

### Outcome measures

The primary outcomes consisted of GM diversity (including α diversity and β diversity) and differences of GM abundance between the patients with AD spectrum and HC. The secondary outcomes consisted of the effects of different countries and clinical stages on GM abundance.

### Data extraction

The necessary data of each study regarding the number of participants, age, body mass index, diabetes mellitus, dietary assessments, diversity and abundance of GM, etc. were extracted by CCH and checked by CHC. Median, minimum, maximum, or 95% confidence interval (CI) from 5 studies were estimated from the bar graphs [25–28, 35]. Discrepancies with study criteria or data coding were resolved by debate and consensus.

### Risk of bias assessment

Two authors (CCH and CHC) independently assessed the risk of bias in each included study using the Risk of Bias Assessment Tool for Nonrandomized Studies (RoBANS) [36], which evaluates six possible sources of bias: selection of participants, confounding variables, measurement of exposure, blinding of outcome assessments, incomplete outcome data, and selective outcome reporting. Disagreements were resolved by consensus or by consultation with a third author.

### Effect size calculations

The Comprehensive Meta-Analysis Version 3 software (Biostat Inc., Englewood, NJ, USA) was applied to calculate the effect sizes with a random-effect, inverse-variance weighted model. Postulating that departures from Gaussian distributions were not serious, we used previously reported conversion equations [37] to estimate means and standard deviation from median, maximum and minimum. Hedges’ g effect sizes were derived from the mean differences between groups of AD spectrum and HC, divided by the pooled standard deviation of these groups. Heterogeneity across each study was evaluated using Q-statistic and I^2^. Additionally, the inclusion of outliers may result in bias and significantly influence the pooled effect sizes [38, 39]. We defined the outliers with the following criteria: 1) for which the upper boundary of the 95% CI is lower than the lower boundary of the overall effect CI (i.e., extremely small effect sizes); (2) for which the lower boundary of the 95% CI is higher than the upper boundary of the overall effect CI (i.e., extremely large effect sizes).

Potential publication bias of each GM abundance was quantitatively assessed by Begg and Mazumdar rank correlation [40] and Egger’s regression intercept tests [41]. Moreover, the Duval and Tweedie’s trim and fill method was used to correct for non-normal distribution of effect sizes potentially due to the file drawer problem. The significant levels were set at *p* < 0.05.

### Availability of data

Data available on request from the corresponding authors.

## RESULTS

### Study selection and characteristics

**Figure 1:**
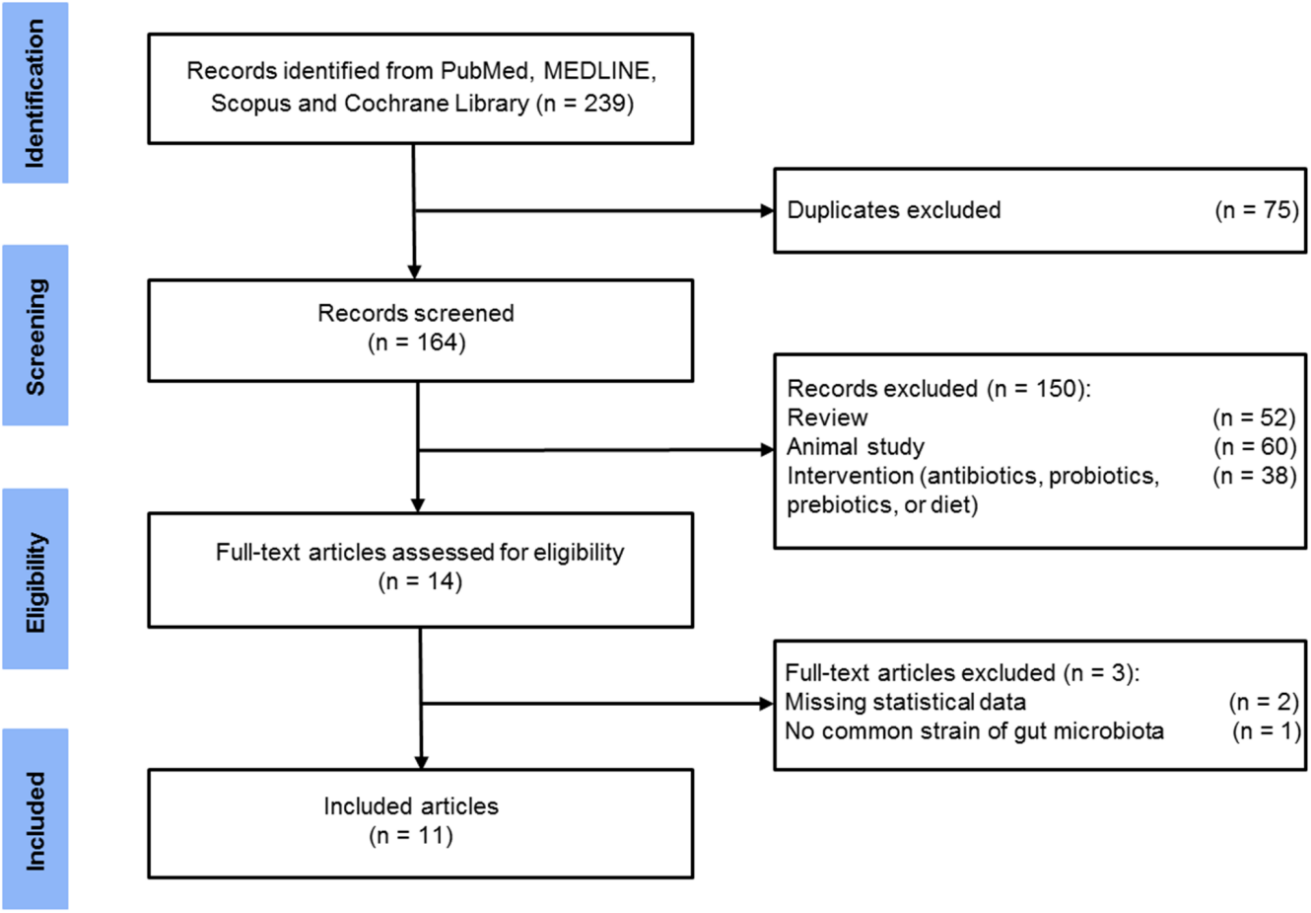
Flow diagram of selected studies.

By the comprehensive literature search, 164 relevant articles were yielded when duplications were excluded. After the review of the titles and abstracts, 14 studies were potentially eligible for our meta-analysis. After carefully examining the full texts, three additional studies were removed: two did not provide sufficient data [42, 43] and one did not report common GM strains as other studies [44]. Therefore, the remaining 11 articles were included in the final meta-analysis (Figure 1). Table 1 summarizes the clinical and demographic characteristics of the 11 studies. These studies were performed in China [23–25, 27, 29, 35, 45, 46] and U.S. [26, 28, 47], with a total of 378 HC and 427 patients with AD spectrum (AD = 251, MCI = 124, aMCI = 52).

**Table 1:** Characteristics of each study included in the meta-analysis.

**Study Country**		**HC**					**Stage**	**AD spectrum**					**Dietary check**	**Genetic analysis**
		**N**	**Sex (M/F)**	**Age**	**BMI**	**DM^a^**		**N**	**Sex (M/F)**	**Age**	**BMI**	**DM^a^**		
Vogt et al. (2017)	U.S.	25	7/18	69.3 ± 7.5	26.1 [24.3, 33.2]^b^	2 (8)	AD	25	8/17	71.3 ± 7.3	26.0 [22.9, 29.1]^b^	2(8)	NR	16S rRNA gene sequencing using Illumina DNA extraction method: NR MiSeq platform (2 × 250-bp) Region: V4 Pipeline analysis: Mothur Database: Greengenes
Zhuang et al. (2018)	China	43	23/20	69.7 ± 9.2	NR	5 (11.6)	AD	43	23/20	70.1 ± 8.8	NR	7 (16.3)	NR	16S rRNA gene sequencing using Illumina DNA extraction method: Power Soil Kit MiSeq platform (2 x 300-bp) Region: V3-V4 Pipeline analysis: QIIME Database: RDP
Haran et al. (2019)	U.S.	51	8/43	80.3 ± 10.2	NR	11 (21.6)	AD	24	4/20	84.7 ± 8.1	NR	5 (20.8)	Yes	DNA extraction method: PowerMag soil DNA isolation kit NextSeq 500 sequencing system (2 x 150-bp) Region: NR Pipeline analysis: KneadData Database: NCBI bacterial genomes k-mer
Li et al. (2019)	China	30	13/17	63.9 ± 5.1	24.0 ± 2.9	2 (6.7)	MCI AD	30 30	12/18 15/15	65.4 ± 7.6 66.3 ± 5.1	23.2 ± 2.9 23.0 ± 3.5	3 (10) 2 (6.7)	NR	16S rRNA gene sequencing using Illumina DNA extraction method: QIAamp DNA Stool Mini Kit MiSeq platform (2 x 300-bp) Region: V3-V4 Pipeline analysis: NR Database: NR
Liu et al. (2019)	China	32	16/16	76.9 ± 9.4	22.2 ± 2.3	1 (3.1)	MCI^c^ AD	32 33	14/18 19/14	70.5 ± 11.0 74.9 ± 11.4	22.4 ± 2.6 22.0 ± 1.3	3 (9.4) 7 (21.2)	NR	16S rRNA gene sequencing using Illumina DNA extraction method: DNA extraction kit MiSeq platform Region: V3-V4 Pipeline analysis: QIIME Database: Greengenes
Nagpal et al. (2019)	U.S.	6	2/4	65.2 ± 3.7	NR	NR	MCI	11	3/8	64.3 ± 7.7	NR	NR	NR	16S rRNA gene sequencing using Illumina DNA extraction method: QiaAmp PowerFecal DNA kit MiSeq platform (2 x 300-bp) Region: V4 Pipeline analysis: QIIME Database: Greengenes
Hou et al. (2021)	China	47	22/25	71.7 ± 6.7	23.6 ± 3.3	3 (6.8)	AD	30	17/13	71.9 ± 6.9	23.7 ± 4.8	7 (23.3)	Yes	16S rRNA gene sequencing using Illumina DNA extraction method: E.Z.N.A Stool Extraction Kit MiSeq platform (2 x 300-bp) Region: V3-V4 Pipeline analysis: UPARSE Database: Greengenes
Liu et al. (2021)	China	22	9/13	72.7 ± 8.05	22.1 ± 2.3	1 (4.5)	MCI^c^	20	12/8	68.8 ± 11.2	22.8 ± 2.3	2 (10)	NR	16S rRNA gene sequencing using Illumina DNA extraction method: DNA extraction kit MiSeq platform Region: V3-V4 Pipeline analysis: QIIME Database: Greengenes
Sheng et al. (2021)	China	38	15/23	66.8 ± 5.1	24.0 ± 3.3	3 (7.9)	CI^d^	14	4/10	73.2 ± 7.9	23.4 ± 3.0	3 (21.4)	NR	16S rRNA gene sequencing using Illumina DNA extraction method: QIAamp DNA Stool Mini Kit MiSeq platform (2 x 300-bp) Region: V3-V4 Pipeline analysis: NR Database: RDP
Zhang et al. (2021)	China	52	24/28	62.5 ± 4.0	24.2 ± 3.1	NR	MCI	75	36/39	62.0 ± 4.1	24.7 ± 2.9	NR	Yes	16S rRNA gene sequencing using Illumina DNA extraction method: Power Fecal DNA Isolation Kit HiSeq platform Region: V4 Pipeline analysis: QIIME Database: NR
Zhou et al. (2021)	China	32	14/18	71.1 ± 5.9	21.7 ± 1.5	4 (12.5)	AD	60	24/36	72.8 ± 7.3	22.1 ± 1.7	10 (16.7)	NR	16S rRNA gene sequencing using Illumina DNA extraction method: QIAamp DNA Stool Mini Kit MiSeq platform (2 x 250-bp) Region: V3-V4 Pipeline analysis: Mothur Database: RDP

Abbreviations: HC: healthy control; MCI: mild cognitive impairment; AD: Alzheimer’s disease; BMI: Body Mass Index; DM: diabetes mellitus; M: male; F: female; NR: not reported; QIIME: Quantitative Insights Into Microbial Ecology; RDP: Ribosomal Database Project. ^a^DM was presented as n (%); ^b^BMI was presented as median [interquartile range]; ^c^amnestic MCI; ^d^the patients consisted of MCI (*n* = 8) and AD (*n* = 6).

### Primary outcomes: α diversity and β diversity

**Figure 2:**
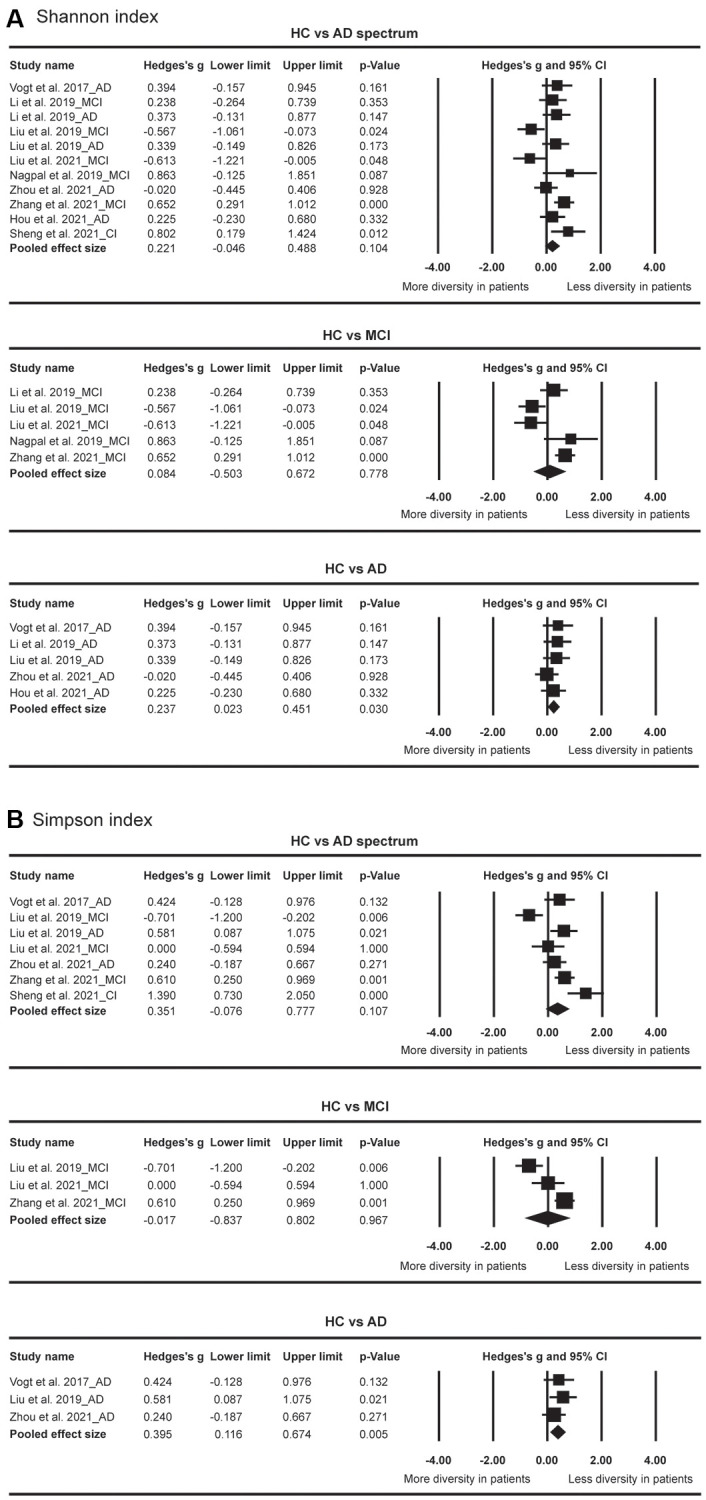
Forest plots of Shannon index (**A**) and Simpson index (**B**) in the comparisons between healthy controls (HC) and Alzheimer’s disease (AD) spectrum. Patients with AD spectrum consisted of mild cognitive impairments (MCI) and AD.

Among the indices of α diversity, Shannon index and Simpson index were most frequently measured in our included articles. There were no significant differences of α diversity between HC and AD spectrum (Figure 2). However, when patients with AD spectrum were divided into those with MCI and AD, the results showed that AD but not MCI demonstrated significantly reduced α diversity as indexed by Shannon index (Hedges’ g = 0.237; 95% CI = 0.023 to 0.451; *p* = 0.030; *n* = 5) and Simpson index (Hedges’ g = 0.395; 95% CI = 0.116 to 0.674; *p* = 0.005; *n* = 3).

Among all the included articles except for three studies [27, 35, 47], seven indicators of β diversity were assessed (Table 2). The principal coordinate analyses based on both Weighted UniFrac distance and Unweighted UniFrac distances were most frequently measured. In terms of Weighted UniFrac distance, three studies revealed significant differences [24, 29, 46], while four studies revealed no significant differences between HC and AD spectrum [23, 25, 28, 45]. In terms of Unweighted UniFrac distances, two studies revealed significances [24, 29] while four studies revealed no significant differences between HC and AD spectrum [23, 25, 28, 45]. In brief, the findings were inconsistent in our included studies.

**Table 2:** Summary of beta diversity assessments in the included studies.

**Study**	**β diversity**	**Findings**	**Statistic value**
Vogt et al. (2017)	NMDS of Weighted UniFrac distances NMDS of Unweighted UniFrac distances NMDS based on Bray-Curtis dissimilarity	A significant difference in gut microbial composition between AD and HC A significant difference in gut microbial composition between AD and HC A significant difference in gut microbial composition between AD and HC	*p* < 0.001 *p* < 0.005 *p* < 0.001
Li et al. (2019)	PCoA of Weighted UniFrac distances PCoA of Unweighted UniFrac distances	A significant difference in gut microbial composition among AD, MCI and HC No significant difference in gut microbial composition between AD and MCI A significant difference in gut microbial composition among AD, MCI and HC No significant difference in gut microbial composition between AD and MCI	*p* = 0.001 NR *p* = 0.001 NR
Liu et al. (2019)	PCoA of Weighted UniFrac distances PCoA of Unweighted UniFrac distances PCoA based on Bray-Curtis dissimilarity	No significant difference in gut microbial composition among AD, MCI^a^ and HC No significant difference in gut microbial composition among AD, MCI^a^ and HC A significant difference in gut microbial composition between AD and HC A significant difference in gut microbial composition between AD and MCI^a^ A significant difference in gut microbial composition between MCI^a^ and HC	NR NR *p* = 0.017 *p* = 0.005 *p* = 0.012
Nagpal et al. (2019)	PCoA of Weighted UniFrac distances PCoA of Unweighted UniFrac distances	No significant difference between MCI and HC No significant difference between MCI and HC	NR NR
Hou et al. (2021)	PCoA of Weighted UniFrac distances PCoA of Unweighted UniFrac distances PCoA based on Bray-Curtis dissimilarity	No significant difference in gut microbial composition between AD and HC No significant difference in gut microbial composition between AD and HC A slight difference in gut microbial composition between AD and HC	*p* = 0.233 *p* = 0.065 *p* = 0.039
Sheng et al. (2021)	PCoA of Weighted UniFrac distances PCoA of Unweighted UniFrac distances PCoA based on Bray-Curtis dissimilarity	A marginal difference in gut microbial composition between CI^b^ and HC No significant difference in gut microbial composition among CI^b^, SCD and HC A significant difference in gut microbial composition between CI^b^ and HC	*p* = 0.053 NR *p* = 0.047
Zhang et al. (2021)	PCoA of Weighted UniFrac distances	A significant difference in gut microbial composition between MCI and HC	*p* = 0.008
Zhou et al. (2021)	PCoA of Weighted UniFrac distances PCoA of Unweighted UniFrac distances PLS-DA	A significant difference in gut microbial composition between AD and HC A significant difference in gut microbial composition between AD and HC A clear difference in gut microbial composition between AD and HC	*p* = 0.026 *p* < 0.001 NR

Abbreviations: HC: healthy control; MCI: mild cognitive impairment; AD: Alzheimer’s disease; SCD: subjective cognitive decline; ACE: Abundance-based Coverage Estimator; NMDS: Non-metric multidimensional scaling; PCoA: Principal Coordinate Analysis; PLS-DA: Partial Least Squares Discriminant Analysis. ^a^amnestic MCI; ^b^the patients consisted of MCI (*n* = 8) and AD (*n* = 6).

### Primary outcome: overall effect sizes by disease

**Figure 3:**
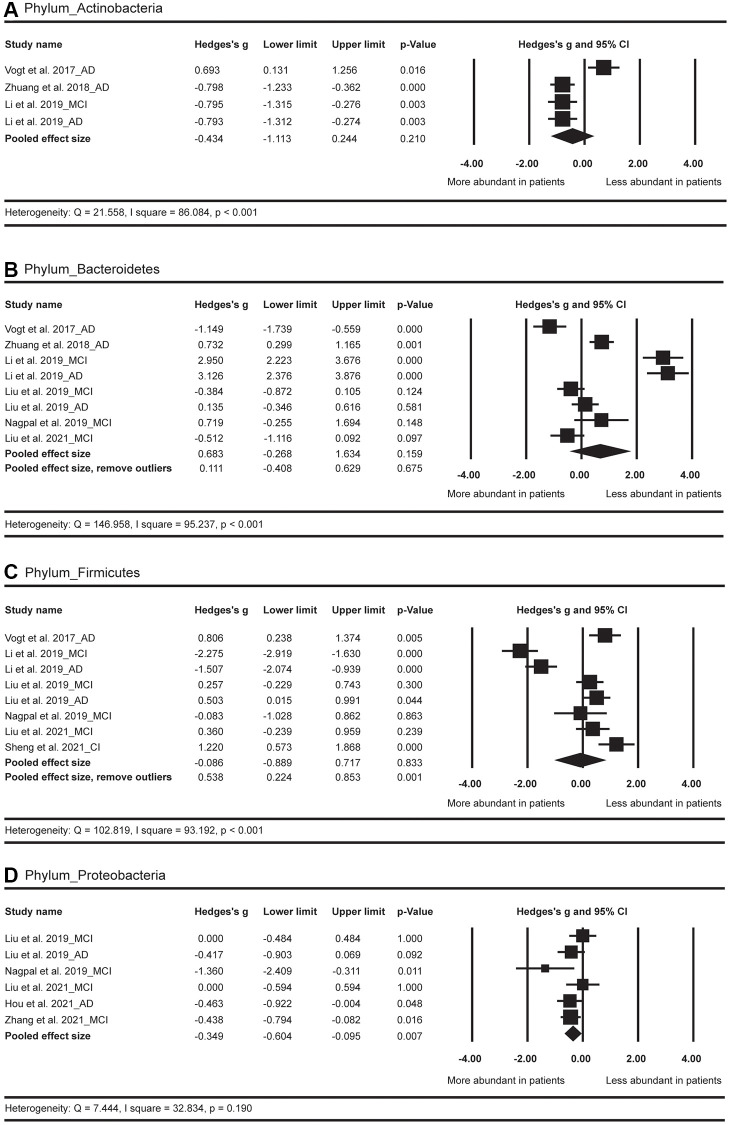
Forest plots of alterations of gut microbiota in the phylum level, including Actinobacteria (**A**), Bacteroidetes (**B**), Firmicutes (**C**), and Proteobacteria (**D**). Abbreviations: AD: Alzheimer’s disease; MCI: mild cognitive impairments; CI: cognitive impairments.

In terms of the phylum level (Figure 3), the results showed more abundance of *Proteobacteria* (Hedges’ g = −0.349; 95% CI = −0.604 to −0.095; *p* = 0.007; *n* = 6) in AD spectrum versus HC. No significant difference was observed for *Firmicutes* between AD spectrum and HC (*p* = 0.833; *n* = 8). After the exclusion of two outliers [24], a significantly less abundance of *Firmicutes* (Hedges’ g = 0.538; 95% CI = 0.224 to 0.853; *p* = 0.001; *n* = 6) was observed in AD spectrum versus HC. The abundance of *Bacteroidetes* and *Actinobacteria* did not show obvious difference between AD spectrum and HC.

**Figure 4:**
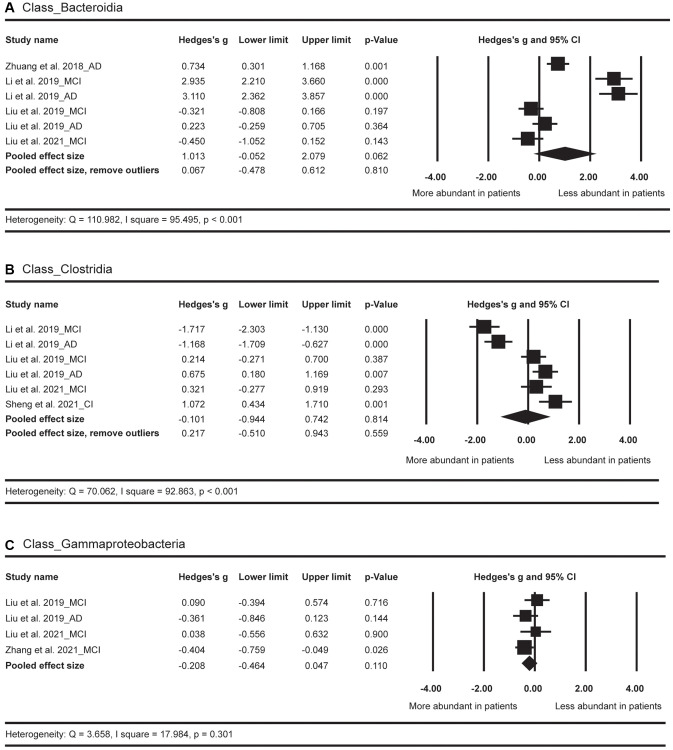
Forest plots of alterations of gut microbiota in the class level, including Bacteroidia (**A**), Clostridia (**B**), and Gammaproteobacteria (**C**). Abbreviations: AD: Alzheimer’s disease; MCI: mild cognitive impairments; CI: cognitive impairments.

In terms of the class level (Figure 4), the abundance of *Bacteroidia*, *Clostridia*, and *Gammaproteobacteria* did not show significant differences between AD spectrum and HC.

**Figure 5:**
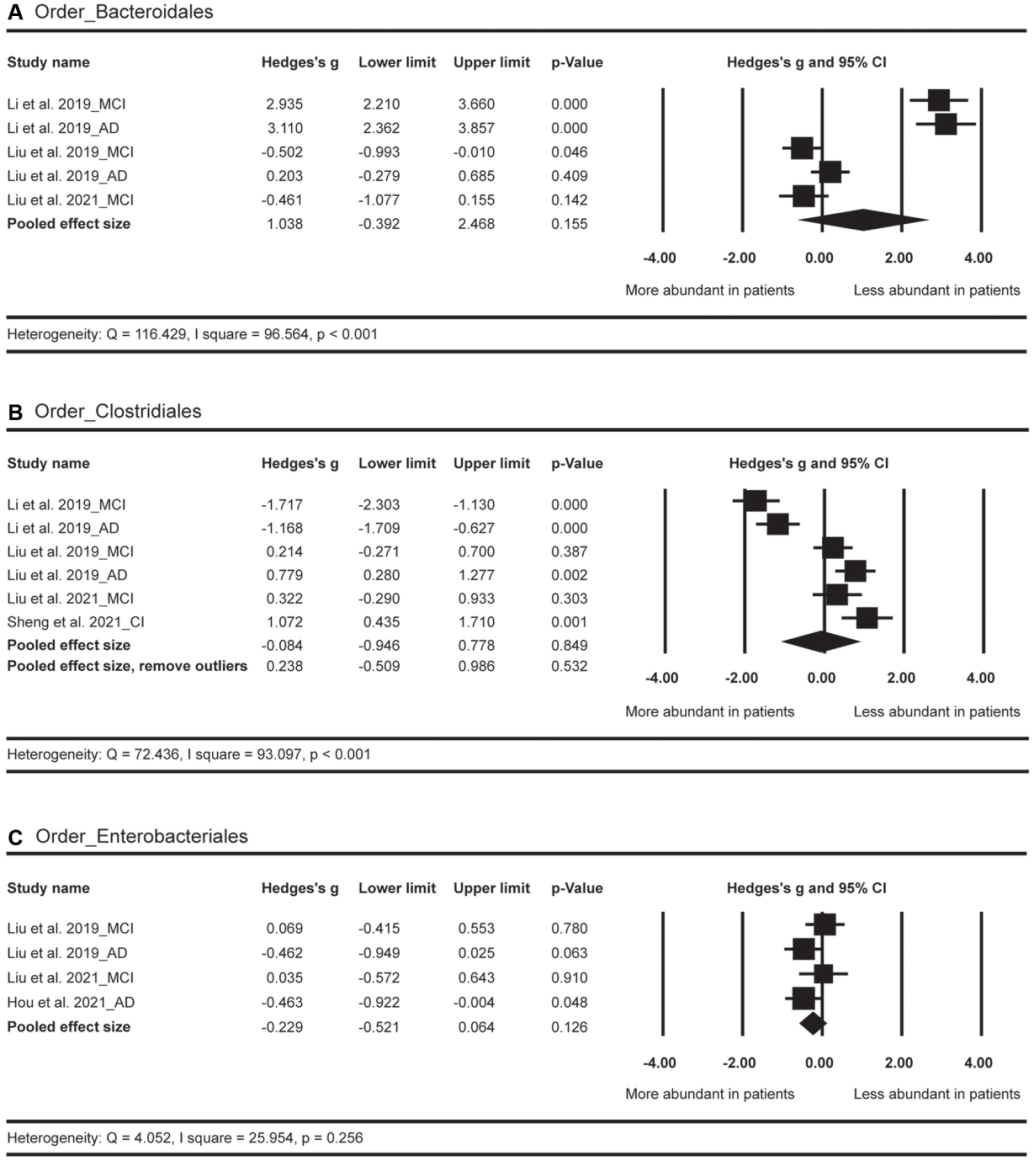
Forest plots of alterations of gut microbiota in the order level, including Bacteroidales (**A**), Clostridiales (**B**), and Enterobacteriale (**C**). Abbreviations: AD: Alzheimer’s disease; MCI: mild cognitive impairments; CI: cognitive impairments.

In terms of the order level (Figure 5), the differences of abundance in *Bacteroidales*, *Clostridiales*, and *Enterobacteriale* were not significant in patients with AD spectrum as compared with HC.

**Figure 6:**
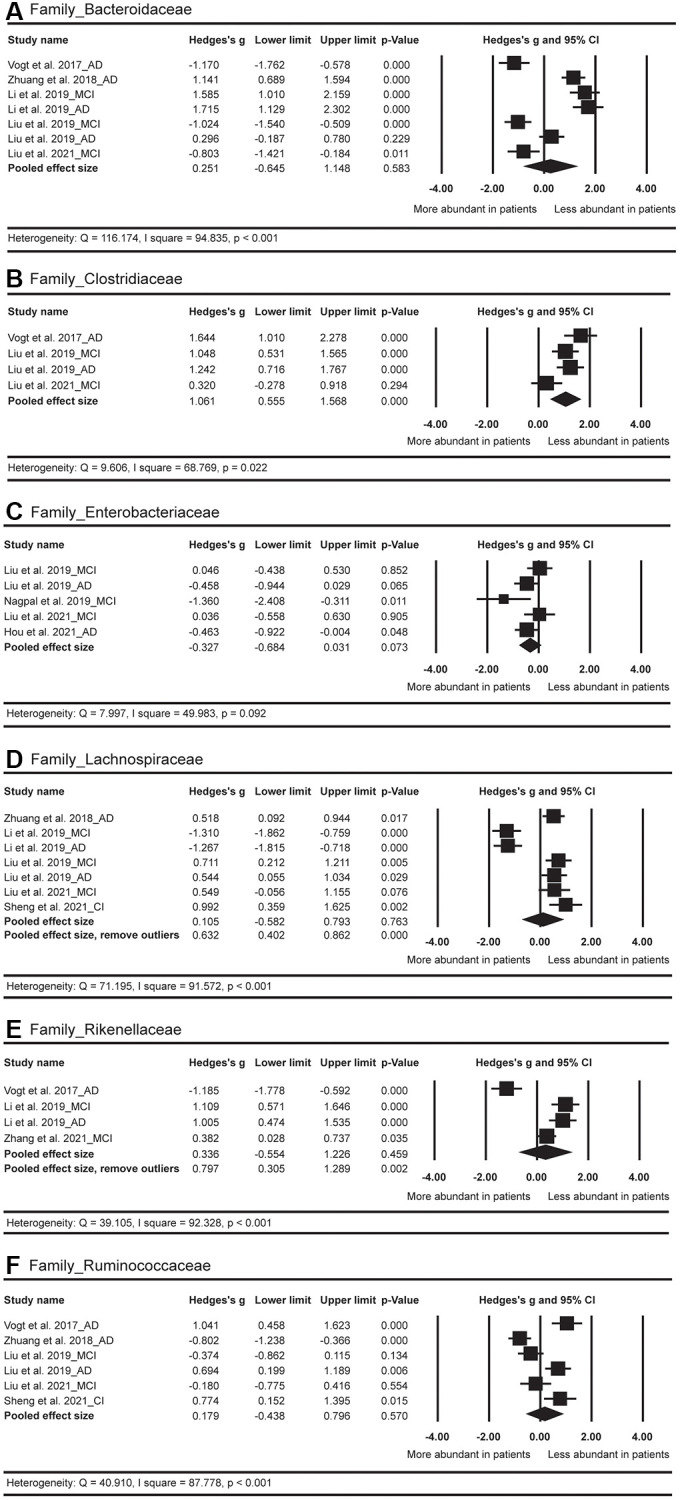
Forest plots of alterations of gut microbiota in the family level, including Bacteroidaceae (**A**), Clostridiaceae (**B**), Enterobacteriaceae (**C**), Lachnospiraceae (**D**), Rikenellaceae (**E**), and Ruminococcaceae (**F**). Abbreviations: AD: Alzheimer’s disease; MCI: mild cognitive impairments; CI: cognitive impairments.

In terms of the family level (Figure 6), the Hedges’ g effect size was 1.061 with 95% CI = 0.555 to 1.568 (*p* < 0.001, *n* = 4) for the *Clostridiaceae*, suggesting a less abundant level of this GM strain in AD spectrum versus HC. The difference of the abundance in *Lachnospiraceae* was not significant between AD spectrum and HC (*p* = 0.763, *n* = 7). After the exclusion of two outliers [24], we discovered a less abundant level of *Lachnospiraceae* (Hedges’ g = 0.632; 95% CI = 0.402 to 0.862; *p* < 0.001; *n* = 5) in AD spectrum versus HC. The abundance of *Rikenellaceae* did not show obvious differences between these two groups (*p* = 0.459; *n* = 4). After the exclusion of one outlier [26], the pooled effect size was 0.797 (95% CI = 0.305 to 1.289; *p* = 0.002; *n* = 3), suggesting less abundant of this GM strain in AD spectrum versus HC. No obvious differences were found for *Bacteroidaceae*, *Enterobacteriaceae*, and *Ruminococcaceae* between these two groups.

**Figure 7:**
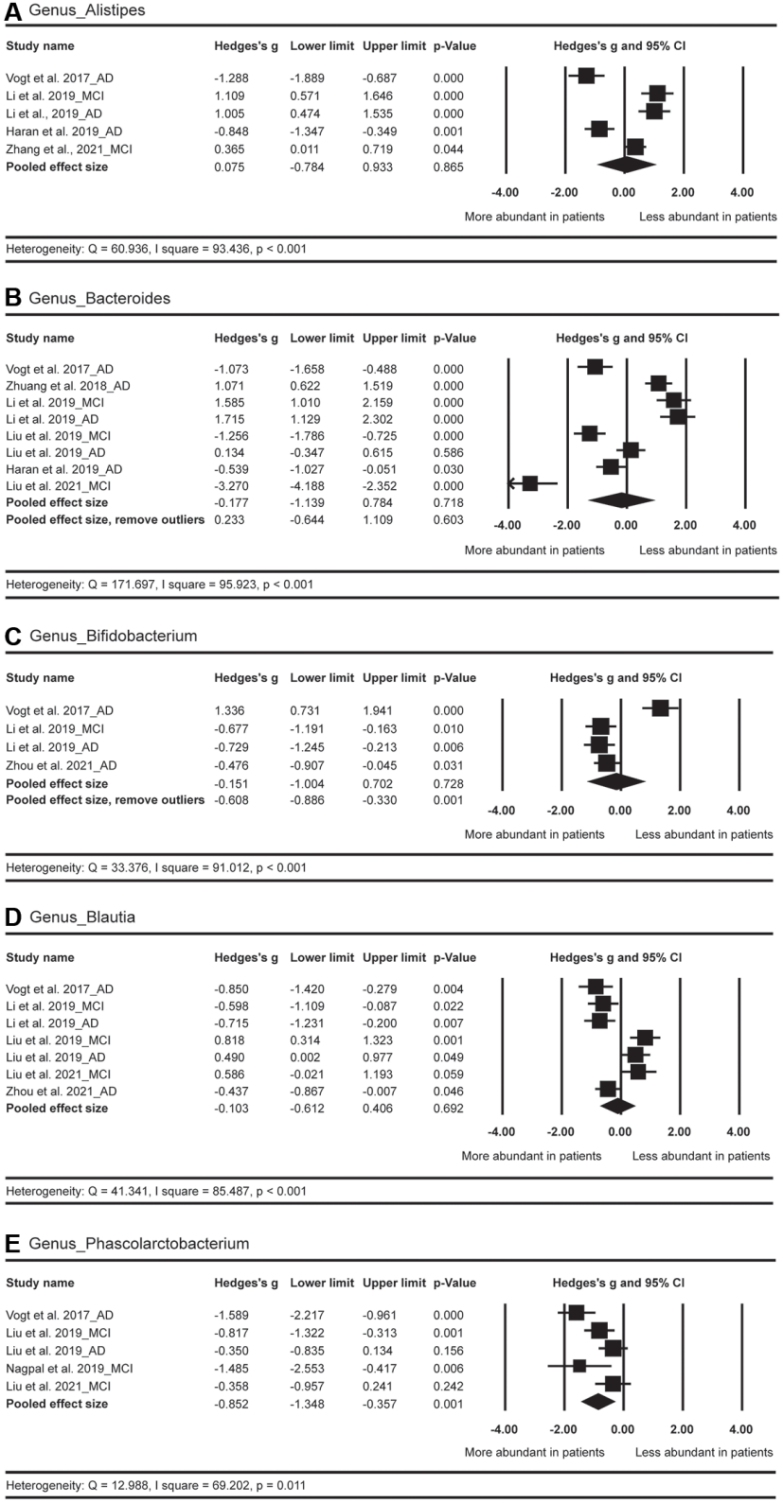
Forest plots of alterations of gut microbiota in the genus level, including Alistipes (**A**), Bacteroides (**B**), Bifidobacterium (**C**), Blautia (**D**), and Phascolarctobacterium (**E**). Abbreviations: AD: Alzheimer's disease; MCI: mild cognitive impairments.

In terms of the genus level (Figure 7), a more abundant level of *Phascolarctobacterium* (Hedges’ g = −0.852; 95% CI = −1.348 to −0.357; *p* = 0.001; *n* = 5) was found in AD spectrum versus HC. The abundance of *Bifidobacterium* did not show obvious differences between AD spectrum and HC (*p* = 0.728; *n* = 4). After the exclusion of one outlier [26], a significantly more abundant level of *Bifidobacterium* (Hedges’ g = −0.608; 95% CI = −0.886 to −0.330; *p* < 0.001; *n* = 3) was detected in AD spectrum versus HC. The abundance of *Alistipes*, *Bacteroides*, and *Blautia* did not show significant differences between these two groups.

### Secondary outcome: effect sizes by country

The pooled effect size for *Bacteroides* was not significant in the comparison between HC and AD spectrum (Figure 7). However, when country was considered as a moderator, the pooled effect sizes for U.S. (*n* = 2) and for China (*n* = 6) were −0.781 (with 95% CI from −1.301 to −0.26, *p* = 0.003) and 0.027 (with 95% CI from −1.194 to 1.249, *p* = 0.965), respectively. In brief, compared with HC, the American patients with AD spectrum showed more abundance of *Bacteroides*, but such as pattern was not found for the Chinese patients (Table 3).

**Table 3:** Summary of effect sizes with 95% CI when country is considered as a moderator.

	**U.S.**			**China**		
	**Hedges’ g**	**95% CI**	***p***	**Hedges’ g**	**95% CI**	***p***
P_Bacteroidetes	−0.257	[−2.086, 1.572]	0.783	0.983	[−0.108, 2.075]	0.077
P_Firmicutes	0.455	[−0.411, 1.301]	0.308	−0.237	[−1.231, 0.758]	0.641
G_Alistipes	−1.035	[−1.461, −0.609]	< 0.001	0.792	[0.287, 1.296]	0.002
G_Bacteroides	−0.781	[−1.301, −0.260]	0.003	0.027	[−1.194, 1.249]	0.965
G_Phascolarctobacterium	−1.562	[−2.104, −1.021]	< 0.001	−0.519	[−0.828, −0.211]	0.001

Abbreviations: CI: confidence interval; P: phylum; G: Genus. The effect sizes were reported only when the number of investigations ≧ 2 in both countries.

It was also interesting to note that in terms of *Alistipes*, the effect size for U.S. was −1.035 (with 95% CI from −1.461 to −0.609, *p* < 0.001; *n* = 2), suggesting more abundant of this GM strain for American patients with AD spectrum. However, the overall effect size for China was 0.792 (with 95% CI from 0.287 to 1.296, *p* = 0.002; *n* = 3), suggesting less abundant of this GM strain for Chinese patients with AD spectrum (Table 3).

Different countries did not significantly modulate the abundance of *Bacteroidetes*, *Firmicutes,* and *Phascolarctobacterium* in the comparisons between HC and AD spectrum.

### Secondary outcome: Effect sizes by clinical stage

The abundance of *Proteobacteria* was increased in the patients with AD spectrum. However, the significance was only found in the comparison between HC and AD (Hedges’ g = −0.441, 95% CI = −0.775 to −0.108, *p* = 0.01; *n* = 2), but not in the comparison between HC and MCI (Hedges’ g = −0.317, 95% CI = −0.739 to 0.106, *p* = 0.142; *n* = 4). Similar trend of abundance was found in the *Phascolarctobacterium*, revealing that the abundance of this GM was significantly increased in patients with MCI versus HC (Hedges’ g = −0.763, 95% CI = −1.277 to −0.248, *p* = 0.004; *n* = 3) (Table 4).

**Table 4:** Summary of effect sizes with 95% CI when clinical stage is considered as a moderator.

	**HC vs. MCI**			**HC vs. AD**		
	**Hedges’ g**	**95% CI**	***p***	**Hedges’ g**	**95% CI**	***p***
P_Bacteroidetes	0.680	[−0.879, 2.239]	0.393	0.693	[−0.712, 2.099]	0.334
P_Firmicutes	−0.434	[−1.683, 0.814]	0.495	−0.063	[−1.438, 1.312]	0.928
P_Proteobacteria	−0.317	[−0.739, 0.106]	0.142	−0.441	[−0.775, −0.108]	0.010
C_Bacteroidia	0.707	[−1.228, 2.642]	0.474	1.323	[−0.087, 2.733]	0.066
C_Clostridia	−0.390	[−1.648, 0.868]	0.544	−0.243	[−2.049, 1.562]	0.792
O_Bacteroidales	0.644	[−1.372, 2.660]	0.531	1.642	[−1.207, 4.490]	0.259
O_Clostridiales	−0.391	[−1.655, 0.874]	0.545	−0.192	[−2.099, 1.716]	0.844
O_Enterobacteriales	0.056	[−0.323, 0.434]	0.773	−0.462	[−0.796, −0.128]	0.007
F_Bacteroidaceae	−0.082	[−1.727, 1.564]	0.922	0.500	[−0.622, 1.621]	0.383
F_Clostridiaceae	0.700	[−0.013, 1.413]	0.054	1.406	[1.001, 1.810]	< 0.001
F_Enterobacteriaceae	0.278	[−0.951, 0.394]	0.417	−0.460	[−0.794, −0.126]	0.007
F_Lachnospiraceae	−0.016	[−1.300, 1.268]	0.980	−0.058	[−1.156, 1.040]	0.917
F_Rikenellaceae	0.716	[0.007, 1.426]	0.048	−0.086	[−2.231, 2.060]	0.937
F_Ruminococcaceae	−0.296	[−0.673, 0.082]	0.125	0.300	[−0.856, 1.456]	0.611
G_Alistipes	0.708	[−0.018, 1.435]	0.056	−0.374	[−1.741, 0.993]	0.592
G_Bacteroides	−0.961	[−3.516, 1.594]	0.461	0.262	[−0.673, 1.197]	0.583
G_Blautia	0.265	[−0.633, 1.162]	0.563	−0.370	[−0.952, 0.212]	0.213
G_Phascolarctobacterium	−0.763	[−1.277, −0.248]	0.004	−0.953	[−2.166, 0.260]	0.124

Abbreviations: CI: confidence interval; P: Phylum; C: Class; O: Order; F: Family; G: Genus. The effect sizes were reported only when the number of investigations ≧ 2 in both diagnoses.

In contrast, we found a trend toward decreased abundance of *Clostridiaceae* in the patients with MCI (Hedges’ g = 0.700, 95% CI = −0.013 to 1.413, *p* = 0.054; *n* = 2), which was more pronounced in the patients with AD (Hedges’ g = 1.406, 95% CI = 1.001 to 1.810, *p* < 0.001; *n* = 2) (Table 4).

### Risk of bias

The quality of the included studies is summarized in Supplementary Table 1. Each study was classified as low risk in five criteria. In the criteria of confounding variables, all studies, except for one [23], suffered from a high-risk bias due to the potential confounding factors (e.g., body mass index, diabetes and diet) on GM structure.

### Publication bias

The Begg and Mazumdar rank correlations as well as Egger’s regression intercept tests confirmed that most of these meta-analysis results were not significantly biased by publication errors. The adjusted Hedges’ g was operated in 4 GM strains, including *Firmicutes*, *Gammaproteobacteria*, *Bacteroidales*, and *Enterobacteriale* (Supplementary Table 2).

## DISCUSSION

This study conducted meta-analysis to compare GM abundance between the patients with AD spectrum and HC, and yielded four major insights into the nature of GM alterations in AD spectrum. First, patients with AD, but not MCI, exhibited decreased GM diversity as compared to HC. Second, *Proteobacteria*, *Bifidobacterium* and *Phascolarctobacterium* were more abundant in AD spectrum, whereas *Firmicutes*, *Clostridiaceae*, *Lachnospiraceae* and *Rikenellaceae* were less abundant in AD spectrum compared to HC. Third, the abundance of *Alistipes* was significantly increased in American patients but significantly decreased in Chinese patients as compared to HC. Altered abundance of *Bacteroides* was only found in the American patients but not in the Chinese patients. Finally, the abundance of *Proteobacteria* and *Phascolarctobacterium* was progressively increased from HC to AD stage, while the abundance of *Clostridiaceae* was gradually reduced from HC to AD stage.

To our knowledge, the present meta-analysis is the first to assess α diversity and β diversity in patients with AD spectrum. Generally, several studies have reported that alpha diversity is significantly decreased in patients with AD [25, 26] but not in patients with MCI [28, 35]. These findings were consistent with our results of meta-analysis, and there was also a trend toward a progressive decline from MCI to AD. Similarly, the decrease of α diversity was also found in other conditions, such as Parkinson’s disease [48] and irritable bowel syndrome (IBS) [49]. In terms of β diversity, further exploration is obviously needed to examine between HC and AD spectrum due to the extremely inconsistent findings.

The *Proteobacteria* is a major phylum of gram-negative bacteria [50]. Of note, the *Proteobacteria* member *Escherichia coli*-derived neurotoxins are correlated with AD neuropathology and increase the release of pro-inflammatory cytokines [44]. It has also been shown that an increased level of *Proteobacteria* was associated with pro-inflammatory dietary pattern (e.g., high-fat diet), and the abundance of *Proteobacteria* increased along with worse memory dysfunction [51, 52]. Taken together, our current finding that patients with AD spectrum showed abnormally more abundance of *Proteobacteria* was supported by previous literature.

The phylum *Firmicutes* serves a connection with inflammatory effects, the modulation of metabolic function and the production of SCFAs [53, 54]. Several lines of evidences have demonstrated that decreased *Firmicutes* was associated with the development of obesity and type 2 diabetes [55, 56]. It was also important to note that insulin resistance might lead to cerebral glucose hypometabolism and enhanced Aβ accumulation in asymptomatic middle-aged people with increased risk of AD [57, 58]. Furthermore, the abundance of *Firmicutes* was positively associated with the performance of executive function, suggesting that it is a kind of beneficial GM strain for the humans [45, 59].

The family *Clostridiaceae* performs a vital function on producing SCFAs, which can offer fuel sources for the host and protective effects on permeability of gut and BBB [60]. Moreover, it has been shown that *Clostridiaceae* was the producers of indole-3-propionic acid, which could prevent oxidative injuries of primary neurons from Aβ [61]. Decrease of *Clostridiaceae* may impair cognitive function and intrinsic brain activities as evident by the results of resting-state functional magnetic resonance imaging [35]. Correspondingly, the family *Lachnospiraceae* is the producer of butyrate, which participates in anti-inflammatory reactions, and in turn maintains the gut barrier [62, 63]. A number of studies have discovered that less abundance of *Lachnospiraceae* would result in insulin resistance, disruptions of homeostasis within the CNS and exacerbation of AD neuropathology [55, 64]. Hence, the *Lachnospiraceae* was considered as an advantageous GM strain.

The genus *Bifidobacterium* is involved in the production of acetate and γ-aminobutyric acid, which has neuroprotective effects on the hosts [65, 66]. It is worth mentioning that *Bifidobacterium* members have been associated with anti-inflammatory effects and reduced permeability of gut [67]. In addition, animal studies have shown that *Bifidobacterium* obviously alleviated the development of AD pathologies [3]. It has also been reported that probiotics with *Bifidobacterium* ameliorated cognitive impairments in patients with AD [68]. However, our meta-analysis exhibited an unexpected result that an increase of *Bifidobacterium* was found in subjects with AD spectrum. This finding might imply the potential gut-brain self-preventative mechanism to rebuild intestinal homeostasis [69]. Nevertheless, it merits future research with a larger sample size to have an in-depth investigation.

The composition and abundance of GM may be influenced by many factors such as age, geographical areas, dietary pattern, and chronic stress. In general, the random-effect sizes for *Bacteroides* and *Alistipes* in AD spectrum did not show obvious differences as compared with HC. These non-significant results were potentially owing to large heterogeneities among the included studies. When considering the country (i.e., China and U.S.) as a moderator, our meta-analysis demonstrated an overgrowth of *Bacteroides* and *Alistipes* in American patients; however, this pattern was not observed in Chinese patients. A previous meta-analysis study has found that Chinese patients with IBS did not show obvious changes of abundance in *Bacteroides* compared to HC; inversely, enriched abundance of *Bacteroides* was observed in patients with IBS from other areas such as U.S. and Finland [49]. It has also been reported that enhanced *Bacteroidetes* (genus *Bacteroides*) members might be a possible signature for AD spectrum since certain members of this phylum are opportunistic pathogens, especially *Bacteroides fragilis* (*B. fragilis*) [70]. Interestingly, *B. fragilis* can be divided into two strains: non-toxigenic *B. fragilis* (NTBF) and enterotoxigenic *B. fragilis* (ETBF). NTBF participates in suppression of colitis and strengthening gut barrier; in contrast, ETBF secretes *B. fragilis* toxins and is highly associated with inflammatory bowel disease [71, 72]. Up to the present, there has been no reasonable interpretation to account for an increased level of *Alistipes* abundance in patients with AD spectrum. It will be a valuable issue for future studies to validate our meta-analysis finding.

We considered the clinical stage as another moderator in the present study. Compared to HC, *Proteobacteria*, and *Phascolarctobacterium* were gradually enhanced from MCI to AD stage. The pro-inflammatory *Proteobacteria* has been suggested as a predictor for AD pathogenesis [23, 35]. In contrast, *Clostridiaceae* was found to be progressively reduced from MCI to AD patients. The abundance of beneficial *Clostridiaceae* has been reported to be significantly decreased in patients with AD, suggesting that it is a distinctive biomarker in predicting the development of AD. In addition, the results of some GM strains in the comparisons of HC versus MCI and HC versus AD did not show gradient changes. The abundance of *Enterobacteriales* was significantly increased in patients with AD, but a little decreased in patients with MCI. This non-gradient pattern from HC to AD was in line with a previous study [25, 35]. The abundance of *Rikenellaceae* was significantly reduced in patients with MCI, but a little enhanced in AD patients, suggesting that the association between *Rikenellaceae* and AD pathogenesis needs further exploration.

In spite of these interesting findings, our study was not without limitations. First, the generalization of these results to other populations is questionable because the vast majority of included studies originated from just two countries. Second, many of the studies suffer from significant sources of bias. There were obvious statistical heterogeneities among the included studies, which could be attributed to differences in dietary pattern, geographical background, center settings, and inclusion criteria of AD spectrum including various regimens, medication doses, illness duration, etc. For example, only three studies [23, 46, 47] in our meta-analysis conducted the dietary assessments. Nevertheless, we applied the random-model to estimate the effect sizes to reduce the influences of the heterogeneities on our results. Third, we manually extracted the necessary data from the bar graphs in several studies, which might lead to another type of bias. However, this procedure was performed by two reviewers with sufficient discussion and consensus. Hence, we reasoned that the direction of the statistical significance in the between-group comparisons would not be substantially affected since we performed this method uniformly across the studies. Fourth, the effects in many occasions were assessed by very few studies and thus the current results should be interpreted with cautions. It merits future research to include more studies to provide stronger evidence on this issue. Fifth, different methods of nucleic acid extraction and gene sequencing (Table 1) are also the potential biases on the results. For example, the differences of GM diversity between HC and AD spectrum might be greater based on the V3-V4 region than those on the V4 region. However, the limited number of studies (three with V4 region and seven with V3-V4 region) impeded us to perform additional analysis.

In conclusion, we demonstrated that *Proteobacteria*, *Bifidobacterium* and *Phascolarctobacterium* were significantly higher abundant in patients with AD spectrum, whereas *Firmicutes*, *Clostridiaceae*, *Lachnospiraceae* and *Rikenellaceae* were significantly lower in patients with AD spectrum compared to HC. Moreover, the dysbiosis of these GM can be viewed as an environmental factor of the AD initiation and progression. In the future, a larger cohort study is needed to further examine the differences of GM in AD spectrum.

## Supplementary Materials

Supplementary Tables
