# Supplementary material for: Gut microbiota in patients with Alzheimer’s disease spectrum: a systematic review and meta-analysis
Source: Aging (Albany NY). 2022 Jan 14;14(1):477–96. doi: 10.18632/aging.203826 (PMC8791218; doi:10.18632/aging.203826)
Supplement: Supplementary Tables [file aging-14-203826-s001.pdf]

SUPPLEMENTARY TABLES

Supplementary Table 1. Bias of the 11 studies included in this meta-analysis based on RoBANS.

Study	The selection of participants	Confounding variables	Measurement of exposure	Blinding of outcome assessments	Incomplete outcome data	Selective outcome reporting
Vogt et al. (2017)	Low risk	High risk	Low risk	Low risk	Low risk	Low risk
Zhuang et al. (2018)	Low risk	High risk	Low risk	Low risk	Low risk	Low risk
Haran et al. (2019)	Low risk	High risk	Low risk	Low risk	Low risk	Low risk
Li et al. (2019)	Low risk	High risk	Low risk	Low risk	Low risk	Low risk
Liu et al. (2019)	Low risk	High risk	Low risk	Low risk	Low risk	Low risk
Nagpal et al. (2019)	Low risk	High risk	Low risk	Low risk	Low risk	Low risk
Hou et al. (2021)	Low risk	Low risk	Low risk	Low risk	Low risk	Low risk
Liu et al. (2021)	Low risk	High risk	Low risk	Low risk	Low risk	Low risk
Sheng et al. (2021)	Low risk	High risk	Low risk	Low risk	Low risk	Low risk
Zhang et al. (2021)	Low risk	High risk	Low risk	Low risk	Low risk	Low risk
Zhou et al. (2021)	Low risk	High risk	Low risk	Low risk	Low risk	Low risk

Abbreviation: RoBANS: Risk of Bias Assessment tool for Non-randomized Studies.

Supplementary Table 2. Publication bias assessments.

	Begg and Mazumdar rank correlation		Egger's regression intercept test		Duval and Tweedie's trim and fill	
	Tau	*P* value	Intercept	*P* value	Observed Hedges' g	Adjusted Hedges' g
Phylum-Actinobacteria	0.500	0.308	15.608	0.353		
Phylum_Bacteroidetes	0.178	0.536	7.657	0.301		
Phylum_Firmicutes	0	1.000	−3.536	0.683	−0.086 [−0.889, 0.717]	−0.272 [−1.068, 0.524]
Phylum_Proteobacteria	0	1.000	−1.324	0.526		
Class_Bacteroidia	0.266	0.452	14.817	0.131		
Class_Clostridia	0.133	0.707	−3.045	0.857		
Class_Gammaproteobacteria	0.167	0.734	3.750	0.265	−0.208 [−0.464, 0.047]	−0.292 [−0.568, −0.017]
Order_Bacteroidales	0.500	0.220	21.963	0.081	1.038 [−0.392, 2.468]	0.530 [−0.881, 1.941]
Order_Clostridiales	0.133	0.707	−2.684	0.883		
Order_Enterobacteriale	0.167	0.734	5.490	0.448	−0.229 [−0.521, 0.064]	−0.334 [−0.643, −0.025]
Family_Bacteroidaceae	0	1.000	−8.140	0.633		
Family_Clostridiaceae	0.167	0.734	0.539	0.970		
Family_Enterobacteriaceae	−0.100	0.806	−2.793	0.349		
Family_Lachnospiraceae	0	1.000	−4.253	0.717		
Family_Rikenellaceae	0	1.000	−2.688	0.819		
Family-Ruminococcaceae	0.400	0.259	13.807	0.139		
Genus_Alistipes	0	1.000	−4.501	0.686		
Genus_Bacteroides	−0.178	0.536	−12.118	0.245		
Genus_Bifidobacterium	0	1.000	16.626	0.309		
Genus_Blautia	−0.285	0.367	2.562	0.817		
Genus_Phascolarctobacterium	−0.500	0.220	−3.835	0.326		

Note: the observed and adjusted effects sizes were reported only when missing studies were found and corrected by Duval and Tweedie's trim and fill. The Hedges' g was presented as overall effect size [lower limit, upper limit].
